# Case Report: Sarcoid-Like Reactions and Tertiary Lymphoid Structures Following Dual Checkpoint Inhibition in a Patient with Early-Stage Lung Adenocarcinoma

**DOI:** 10.3389/fimmu.2022.794217

**Published:** 2022-01-31

**Authors:** Xiaoliang Zhao, Dongsheng Yue, Juanjuan Qian, Lei Zhang, Jin Song, Bin Zhang, Chunmei Zhang, Leina Sun, Yuchen Ma, Henghui Zhang, Changli Wang

**Affiliations:** ^1^ Tianjin Medical University Cancer Institute and Hospital, Tianjin Key Laboratory of Cancer Prevention and Therapy, National Clinical Research Center for Cancer, Tianjin, China; ^2^ Department of Medicine, Genecast Biotechnology Co., Ltd, Wuxi, China; ^3^ Beijing Shijitan Hospital, and School of Oncology, Capital Medical University, Beijing, China

**Keywords:** sarcoid-like reaction, tertiary lymphoid structure, immune checkpoint inhibitor, tumor immune microenvironment, non-small cell lung cancer

## Abstract

Immune checkpoint inhibitor-induced sarcoid-like reactions and tertiary lymphoid structures (TLSs) are increasingly recognized but rarely reported in the same patient. We report a patient with lung adenocarcinoma who displayed sarcoid-like reactions in intrathoracic lymph nodes and tertiary lymphoid structures in surgical tumor after neoadjuvant therapy with nivolumab plus ipilimumab. Pathological examination revealed 50% residual tumor cells after treatment, and the CT evaluation of the primary tumor showed a stable disease. The patient experienced a recurrence eight months after surgery. To identify immune correlates of the limited response to immunotherapy, we conducted genomic and transcriptional assays, multiplex immunoassay, and multiplex immunohistochemistry on the pre- and post-immunotherapy tumor, lymph node, and plasma samples. *TP53* R181C, *KRAS* G12C and *SMAD4* R361H were identified as driver mutations of the tumor. In addition to abundant infiltrated lymphocytes, immunotherapy induced high levels of inhibitory components in post-treatment tissue samples, especially the FOXP3^+^ regulatory T cells in tumor and PD-L1 expression in the lymph node. Despite abundant TLSs in the post-treatment tumor, most TLSs were immature. Moreover, increasing levels of circulating checkpoint proteins BTLA, TIM-3, LAG-3, PD-1, PD-L1, and CTLA4 were observed during immunotherapy. Collectively, our observations revealed that high levels of immunosuppressive molecules in tumor, lymph nodes and/or in peripheral blood might indicate poor outcomes after immunotherapy, even in the setting of a patient with concurrent sarcoid-like reactions and tertiary lymphoid structures.

## Introduction

Immune checkpoint inhibitors (ICIs), while significantly improving survival in patients with multiple advanced cancers, are associated with a unique set of immune-related adverse events, including sarcoid-like reactions (SLRs). ICI-induced SLRs have been reported most commonly in patients with melanoma and lung cancer, and occur in intrathoracic locations (lung and/or mediastinal lymph nodes) and the skin (1). SLRs are histologically characterized as non-caseating granulomas without malignant cells. Patients may be asymptomatic or may have no severe manifestations, and the reactions can spontaneously resolve without specific treatment or ICI discontinuation (2, 3). The incidence of ICI-induced SLRs remains unclear as the reaction is easily mistaken for disease progression and clinicians usually have low awareness (4). However, SLRs are attracting increasing attention in the neoadjuvant setting for non-small cell lung cancer (NSCLC) due to their influence on clinical treatment planning of curative surgery. In NEOSTAR study, SLR, which was defined as nodal immune flare, was found in 16% (7/44) of patients with early-stage NSCLC after neoadjuvant ICI therapy (5). Another phase II trial reported that 13% of 15 patients with resectable NSCLC developed SLRs after inductive pembrolizumab monotherapy (6). ICI-induced SLRs have been reported to associate with favorable therapeutic response in patients with melanoma (7, 8), while there is little known about the association of SLRs with immunotherapy outcomes in lung cancer patients.

Tertiary lymphoid structures (TLSs) are ectopic lymphoid aggregates that developed at chronic inflammatory sites in non-lymphoid tissues including tumors (9). Mature TLSs are characterized by a T-cell zone and a germinal center with proliferating B cells. Across a variety of tumors, the presence of TLSs is associated with favorable clinical outcomes, despite several reports describing negative prognostication of TLSs (10, 11). The prognostic value of TLSs in NSCLC has been reported in several studies since a decade ago. The high density of follicular B cells or mature dendritic cells in TLSs, and high density of TLSs, were associated with favorable prognosis in NSCLC patients (12–15). Moreover, B cells and mature TLSs are demonstrated to predict therapeutic efficacy of immunotherapy across different tumor types (16–19), arousing the interest in the artificial induction of TLSs in tumor therapy. And in the post-treatment samples of non-small cell lung cancer, the presence of TLSs with a germinal center was shown to correlate with the pathological response to neoadjuvant anti-PD-1 therapy (20). However, the formation mechanism and antitumor effect of TLSs deserve further exploration, and standardized evaluation methods need to be established before TLSs can be used to guide clinical decisions.

Here we report a stage IB NSCLC patient with SLRs and TLSs induced by neoadjuvant nivolumab plus ipilimumab. We examined the immune microenvironment of the tumor and lymph nodes, as well as the dynamics of immune-related proteins in peripheral blood, to reveal the immune features of the patient and explore correlates of the limited response to immunotherapy.

### Case Presentation

**Figure 1:**
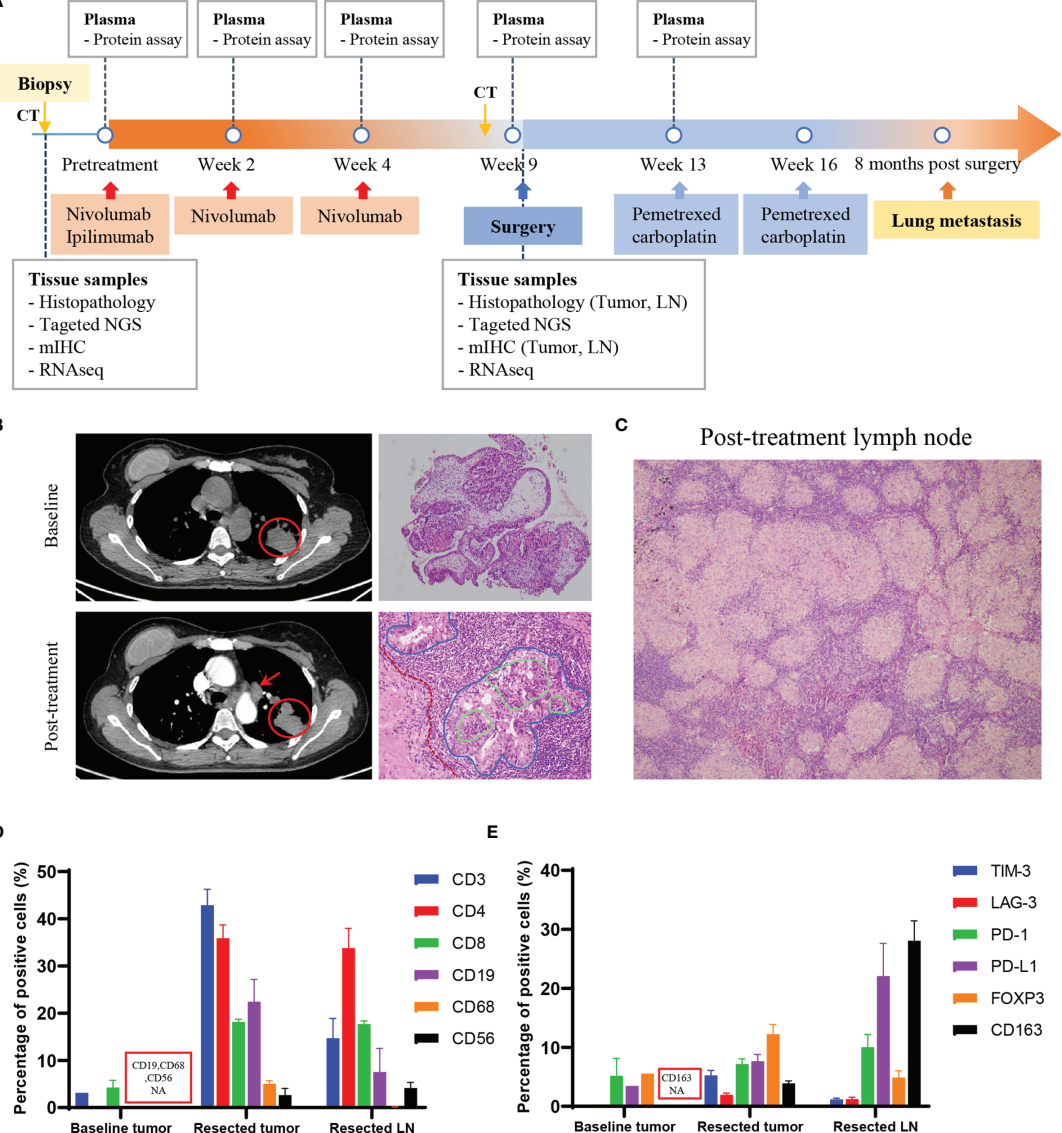
Treatment course of the patient with lung adenocarcinoma. **(A)** Time line of clinical events, along with the time points of sample collection and analyses. **(B)** CT images showed the primary tumor (red circle) and lymph node (red arrow), and the pathological images of the tumor pre- and post-immunotherapy treatment. Red dotted line: fibrosis in the tumor stroma; blue solid line: cancer nest; green solid line: lymphocytes infiltrating into the cancer nest. Magnification: 100×, 200×. **(C)** Pathological image of the post-treatment lymph node showed non-caseating granulomas. Magnification: 100×. **(D, E)** Quantitative results of immune cell markers **(D)** and regulatory or inhibitory markers **(E)** in the baseline biopsy tumor (pre-treatment), resected tumor and resected lymph node (post-treatment) by mIHC assay. For bar graph: *error bar* represents *SEM*. CT, computed tomography; NGS, next-generation sequencing; mIHC, multiplex immunochemistry; LN, lymph node; NA, not available.

A 54-year-old non-smoking Chinese woman was referred to our hospital because of a mass which was incidentally discovered by radiological examination during a routine medical checkup. She had no cough, chest tightness or chest pain, and no other abnormalities were found. The patient reported no history of autoimmune disease or family history of tumor. Positron emission tomography-computed tomography (PET-CT) revealed a 39 mm×45 mm×45 mm mass with abnormally increased intake of 18F-fluorodeoxyglucose. And CT-guided biopsy confirmed adenocarcinoma. The patient was diagnosed with stage IB (cT2N0M0) lung adenocarcinoma in August 2018 (**Figure 1A**). Then she started to receive neoadjuvant immune checkpoint inhibitors (ICIs) nivolumab (3 mg/kg, days 1, 15, 29) plus ipilimumab (1 mg/kg, day 1). No immune-related adverse events were found during immunotherapy. One month after the last dose of nivolumab, CT scan revealed enlargement of the primary tumor and multiple lymph nodes (**Figure 1B**). PET-CT showed that the primary lesion diameter increased by approximately 5% compared with that at baseline, and the standard uptake value (SUV) increased from 7.6 to 10.5. Increased hypermetabolic activity was observed in the superior mediastinal vascular space, mediastinal right brachial vein and posterior vena cava, right pulmonary artery, para-aortic arch, subcarina and both pulmonary hila. Preoperative examination showed that the patient’s cardiopulmonary function was normal and suitable for surgery. One week later, the patient underwent left upper lobectomy and radical lymph node dissection through video-assisted thoracic surgery (VATS). The size of the excised tumor was 45 mm×43 mm×37 mm, and a total of 16 lymph nodes were removed. One month after surgery, the patient started to receive two cycles of routine chemotherapy, pemetrexed plus carboplatin, every 3 weeks. During chemotherapy, the patient experienced persistent radiating and dull pain in the left posterior chest. Aortic dissection (Stanford B) was found on the first postoperative CT scan after chemotherapy and then the patient underwent endovascular stent-graft placement. However, another aortic dissection in the abdominal aorta was found on the second follow-up CT scan three month later and the patient refused surgery. Eight months after surgery, the patient developed a lung metastasis (**Figure S1**) and began to receive treatment at a local hospital.

Histopathological examination of the resected tumor revealed advanced lung adenocarcinoma, a relatively low ratio of viable tumor cells (50%) and large numbers of infiltrating lymphocytes (**Figure 1B**); all resected lymph nodes were negative for metastases but with extensive histiocytic nodular hyperplasia (**Figure 1C**). A diagnosis of sarcoid-like reaction in lymph nodes was made for this patient. Driver mutations associated with tumorigenesis were examined by next-generation sequencing (NGS) (**Supplementary Methods**). *TP53* R181C, *KRAS* G12C and *SMAD4* R361H were identified, with the mutant allele frequency (MAF) of 6.9%, 5.5% and 6.2% in the resected tumor and 26%, 23.4% and 34.4% in the tissue obtained by tumor biopsy prior to the onset of immunotherapy. *EGFR* L858R was found in the baseline tumor, but the MAF was 0.59%. *ALK* and *ROS1* rearrangements were not found.

### Profiling of Local and Peripheral Immune Characteristics

**Figure 2:**
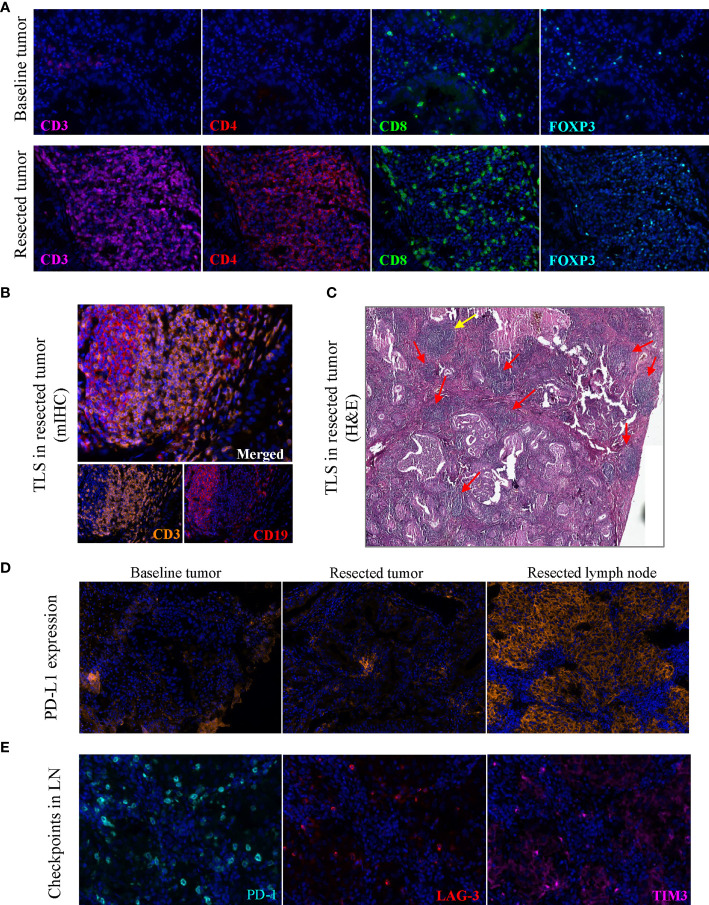
Images of tertiary lymphoid structures and immune cell markers. **(A)** Images showed the abundant CD3^+^, CD4^+^, CD8^+^ and FOXP3^+^ cells in post-treatment tumor on the same slide, with the markers stained on baseline tumor as a contrast. **(B)** TLS in the post-treatment tumor stained by multiplex immunohistochemistry. **(C)** TLSs on a hematoxylin and eosin-stained section, yellow arrow indicates a mature TLS with a pale area, red arrow indicates immature TLSs. **(D)** PD-L1 expression in the baseline tumor, post-treatment tumor and post-treatment lymph node tissue. **(E)** Immune checkpoint proteins PD-1, LAG-3, TIM3 in the post-treatment lymph node tissue. Original magnification of fluorescence image: 200×. TLS, tertiary lymphoid structure; LN, lymph node; mIHC, multiplex immunohistochemistry.

The local immune microenvironment was explored by multiplex immunohistochemistry (mIHC) assay using the Opal seven-color IHC Kit (PerkinElmer, USA). With three staining panels, we analyzed the multiple immune components in the pre- and post- immunotherapy tissue samples, including T lymphocytes (CD3, CD4, CD8), B lymphocytes (CD19), macrophages (CD68), natural killer cells (NK cells, CD56) and a series of regulatory (FOXP3, CD163) or inhibitory (PD1, PD-L1, TIM-3, LAG-3) markers (**Figure S2**). For surgical specimens, more than 10 fields of view in 200× magnification of each tissue slide were selected to calculate percentage of the positive cells in all nucleated cells. The average density of positive cells was shown in **Table S1**. Detailed methods were provided in the **Supplementary Material** (**Supplementary Methods**). The quantitative results showed that the post-treatment tumor and lymph node were infiltrated with a large number of lymphocytes (**Figure 1D**) and rich in high levels of inhibitory molecules or checkpoint proteins (**Figure 1E**). Of note, among the inhibitory markers, the FOXP3^+^ regulatory T cells (Treg) accounted for one-third of helper T cells in tumor tissue (**Figures 1D, E** **)**. Obviously, immunotherapy induced an inflammatory environment in the primary tumor compared to baseline (**Figure 2A**). According to the high infiltration of B lymphocytes, we further observed tertiary lymphoid structures (TLSs) in the post-treatment tumor, characterized by a dense aggregation of CD3^+^ T lymphocytes and CD19^+^ B lymphocytes (**Figure 2B**). We scanned the whole tissue section on a hematoxylin and eosin-stained slide and counted the TLSs (**Figure 2C**). A total of 31 TLSs were found, with a density of 0.27 TLS per mm^2^, accounting for 3.3% of the whole tissue area. However, there were very few TLSs with a germinal center (GC), suggesting that most TLSs were immature. And we observed high expression of checkpoint proteins, especially PD-L1, in lymph node tissue after immunotherapy (**Figures 2D, E**). The expression of PD-L1 in baseline tumor and post-treatment tumor was relatively low, but was abnormally high in the post-treatment lymph node. Based on the immune microenvironment of the tumor and lymph node, it seems difficult to infer whether immunosuppressive factors predominated in the intense combat between immune system and tumor triggered by ICIs.

**Figure 3:**
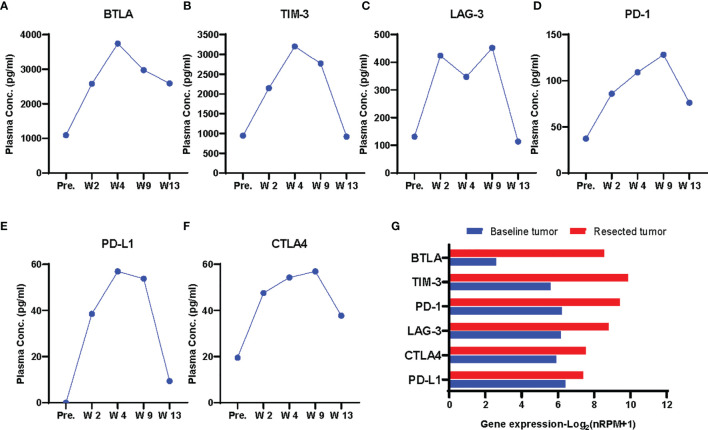
Dynamics of plasma immune checkpoint proteins. **(A–F)** Plasma concentrations of checkpoint proteins BLTA **(A)**, TIM-3 **(B)**, LAG-3 **(C)**, PD-1 **(D)**, PD-L1 **(E)**,CTLA4 **(F)** at each time point. pre., W2, W4, prior to each cycle of immunotherapy; W9, prior to the radical surgery; W13, four weeks after surgery. The proteins were upgregulated during immunotherapy treatment. **(G)** Gene expression of the immune checkpoint proteins in pre- and post-treatment tumor by RNA sequencing.

Then we explored the changes of peripheral immune factors. Blood samples were collected prior to each cycle of immunotherapy and the radical surgery, and one month after surgery as shown in **Figure 1A**. A total of plasma 59 proteins, including cytokines, chemokines, growth factors, and checkpoint proteins, were simultaneous detected by two ProcartaPlex panels with sandwich ELISA based multiplex immunoassays (**Supplementary Methods**). The results show that all detectable checkpoint proteins were increased during the neoadjuvant immunotherapy (**Figure S3**), such as B and T lymphocyte attenuator (BTLA), T cell immunoglobulin and mucin domain-containing protein 3 (TIM-3), lymphocyte-activation gene 3 (LAG-3), programmed cell death 1 (PD-1), programmed cell death ligand 1 (PD-L1), cytotoxic T-lymphocyte antigen 4 (CTLA4) (**Figures 3A–F**). As accumulating evidence shows that these circulating immune checkpoints are associated with a poor response to immune checkpoint blockade, the ascending concentrations of the checkpoint proteins during treatment might suggest activation of alternative immune evasion tactics of tumor. Moreover, we also examined the expression of these proteins in tumor tissue pre- and post- neoadjuvant immunotherapy by RNA sequencing. In consistent with the findings about plasma proteins, RNA expression of the checkpoint proteins was upregulated in the tumor after immunotherapy (**Figure 3G**).

## Discussion

We report the tumor-immune features of an early-stage NSCLC patient with SLRs and TLSs after neoadjuvant nivolumab plus ipilimumab. In addition to abundant infiltrating immune cells, we also found high levels of inhibitory components in post treatment tissue samples, especially the Treg cells in tumor and PD-L1 expression in the lymph node. Despite high number of TLSs, most of them were immature and might not have efficient anti-tumor activity. Moreover, extensively increasing immune checkpoint proteins were found during immunotherapy treatment. Hence, it is suggested that the effect of immunosuppression in this patient is equal or superior to the beneficial antitumor effect induced by ICIs, leading to the limited response to immunotherapy.

It is interesting to find SLRs and TLSs in the same patient treated with ICIs. Although an association of them in immunotherapy setting was suspected, the similar case has not been reported yet. Collectively, the two resections are associated with an inflammatory immune environment, and assumed to correlate with favorable outcomes of melanoma patients treated with immunotherapy (7, 8, 17). However, the patient here didn’t benefit from immunotherapy, with a stable disease evaluated by radiology and 50% residual tumor cells by histology after treatment, and had a recurrence 8 months after surgery. According to the previous case reports of 8 SLR patients with NSCLC (21–28), 4 achieved partial response, 2 had progressive disease, and 2 had stable disease after immunotherapy. The association between SLRs and immunotherapy outcomes in lung cancer seems not clear. On the other hand, although total TLS and germinal center-positive (GC+) TLS subset scores were demonstrated to predict survival in resected NSCLC patients (29), most studies suggested that only TLSs with GC were functional, and B cells in immature TLSs could adopt a regulatory phenotype and inhibit immune reactions (30). Moreover, we noted the predominant inhibitory Treg cells in post treatment tumor and PD-L1 expression in the lymph node for their crucial roles in immunosuppression. Treg cells are one of the well-known cell types that can suppress anti-tumor immune response (31). And tumor-infiltrating follicular regulatory T cells, which are primarily located within TLSs and exhibit superior suppressive capacity and *in vivo* persistence as compared with Treg cells, could impair the survival of patients and impede the efficacy of immunotherapy treatment by regulating TLS (32). Nevertheless, a retrospective study showed that high TLS-B cell density could counterbalance the deleterious impact of high Treg cell density on survival of untreated NSCLC patients (33). It is difficult to fully assess TLSs and Treg cells by biopsy before neoadjuvant immunotherapy, while Treg cells in the posttreatment tumor tissue of this patient were deemed to impair anti-tumor immune response. As for the high level of PD-L1 expression in lymph nodes, which has not been reported in ICI-induced SLR cases, a recent study showed that lymphatic endothelia PD-L1 expression reduced tumor immunity, inducing apoptosis in tumor-specific CD8+ central memory cells in tumor-draining lymph nodes (34). Therefore, we highlight the importance of investigating inhibitory immune components in microenvironment of tumor and lymph nodes when assessing local immune status.

There is accumulating evidence indicating that high levels of circulating immune checkpoint proteins were associated with poor prognosis in a variety of cancers, such as BTLA and TIM-3 in clear cell renal cell carcinoma (35) and PD-1, PD-L1 and BTLA in pancreatic adenocarcinoma (36). The circulating checkpoint proteins also showed a predictive value in ICI-treated patients. LAG-3 expression on pretreatment peripheral blood cells could identify patients with melanoma who may not benefit from immune checkpoint blockade (37). High levels of LAG-3 and PD-1 in pre-treatment serum samples of melanoma patients may predict resistance to anti-PD-1 treatment and anti-PD-1 plus anti-CTLA4 respectively (38). Moreover, increased tumor infiltrated TIM3^+^ or LAG3^+^ T cells also correlated with a shorter progression free survival or adaptive resistance to anti-PD-1 therapy (38, 39).

There are several limitations of this study. First, we only have one patient with co-occurrent SLRs and TLSs who did not respond well to neoadjuvant immunotherapy, so the association between the reactions and inhibitory immune components and clinical outcomes remain to be explored in more patients. Second, we did not determine the comprehensive cellular composition of tertiary lymphoid structures in tumor tissue or the main cell types expressing PD-L1 in lymph nodes due to lack of enough sample.

This report presents the special reactions SLR and TLS and immune characteristics of a NSCLC patient during the treatment with neoadjuvant immune checkpoint inhibitors, which may provide a new perspective for exploring the mechanism of immunotherapy and looking for new predictive markers.

## Data Availability Statement

The original contributions presented in the study are included in the article/**Supplementary Material**. Further inquiries can be directed to the corresponding authors.

## Ethics Statement

The studies involving human participants were reviewed and approved by the ethics committee of Tianjin Medical University Cancer Institute and Hospital. The patients/participants provided their written informed consent to participate in this study. Written informed consent was obtained from the individual(s) for the publication of any potentially identifiable images or data included in this article.

## Author Contributions

Conception and design: CW and HZ. Provision of study materials or patients: XZ and DY. Collection and assembly of data: JS, BZ, CZ, LS, and YM. Analysis and interpretation of data: XZ, DY, and JQ. Manuscript writing and revision: JQ and LZ. All authors contributed to the article and approved the submitted version.

## Funding

This study was supported by the National Key Sci-Tech Special Project of China (2018ZX10302207).

## Conflict of Interest

JQ, JS, LZ, and CZ are employed by Genecast Biotechnology Co., Ltd.

The remaining authors declare that the research was conducted in the absence of any commercial or financial relationships that could be construed as a potential conflict of interest.

## Publisher’s Note

All claims expressed in this article are solely those of the authors and do not necessarily represent those of their affiliated organizations, or those of the publisher, the editors and the reviewers. Any product that may be evaluated in this article, or claim that may be made by its manufacturer, is not guaranteed or endorsed by the publisher.

## Abbreviations

ICI, immune checkpoint inhibitor; SLR, sarcoid-like reaction; TLS, tertiary lymphoid structure; NSCLC, non-small cell lung cancer; PET-CT, positron emission tomography-computed tomography; MAF, mutant allele frequency; mIHC, multiplex immunohistochemistry; NK cell, natural killer cell; Treg cell, regulatory T cell; PD-1, programmed cell death 1; PD-L1, programmed cell death ligand 1; CTLA-4, cytotoxic T-lymphocyte antigen 4; TIM-3, T cell immunoglobulin and mucin domain-containing protein 3; BTLA, B and T lymphocyte attenuator; LAG-3, lymphocyte-activation gene 3; GC, germinal center.
