# Supplementary material for: Case Report: Sarcoid-Like Reactions and Tertiary Lymphoid Structures Following Dual Checkpoint Inhibition in a Patient with Early-Stage Lung Adenocarcinoma
Source: Front Immunol. 2022 Jan 31;13:794217. doi: 10.3389/fimmu.2022.794217 (PMC8841621; doi:10.3389/fimmu.2022.794217)
Supplement: Supplementary file 1 [file DataSheet_1.pdf]

Supplementary Content

Methods

Figure S1. CT image of the lung metastasis.

Figure S2. Representative images of multiplex immunofluorescence of the three staining panels.

Figure S3. Heatmap of the plasma concentrations of 59 immune-related proteins.

Table S1. Quantitative data of positive immune cells stained by multiplex immunohistochemistry.

Table S2. Multiplex immunohistochemical staining protocol.

Methods

DNA sequencing and somatic variant calling

DNA from formalin-fixed paraffin-embedded (FFPE) tumor tissue and peripheral blood lymphocytes was extracted and quantified respectively. DNA libraries were constructed by KAPA HTP Library Preparation Kit (Illumina platforms) (KAPA Biosystems, Massachusetts, USA), and was sequenced by a custom-designed capture-based assay involving 543 cancer-related genes (Genecast Biotechnology Co., Ltd.). The captured libraries were loaded into the NovaSeq 6000 system (Illumina) for 150bp paired-end sequencing. Raw data of generated reads from paired samples (FFPE and peripheral blood sample) were aligned to the human reference genome (hg19) using BWA (Burrows-Wheeler Aligner, v0.7.12). Somatic single nucleotide variants (SNVs) were called using Vardict (v1.5.1) and somatic indels were called using GATK (v3.7). Variants were filtered by a false positive filtering pipeline based on a database of more than 1000 samples. And SNPs were filtered by dbSNP, 1000g and ExAC database (population frequency >0.01).

Multiplex immunochemistry assay

Three 4-μm FFPE tissue slides of each sample (pretreatment tumor, post-treatment tumor, post-treatment lymph node) were used for immunofluorescence staining: slide 1 was stained for FOXP3, CD4, CD8, PD-1, CD3, Pan Cytokeratin (PanCK); slide 2, CD8, TIM-3, PD-1, LAG-3, PD-L1, PanCK; slide 3, CD163, CD68, CD3, CD56, CD19, PanCK. The slides were deparaffinized, rehydrated, and washed, followed by

microwave antigen retrieval in Tris–EDTA (pH 9.0). For every slide, 6 cycles of staining were performed corresponding to each primary antibody. And each cycle comprises microwave treatment, protein blocking, antibody incubation and TSA visualization. Details of conditions are presented in the supplementary document (Supplementary table S1). During the staining after the secondary antibody incubation, incubation with Opal Ploymer HRP Ms+Rb (2414515; PerkinElmer, Massachusetts, USA) was performed at 37°C for 10 min followed by TSA visualization with the Opal seven-color IHC Kit (NEL797B001KT; PerkinElmer) and TSA Coumarin system (NEL703001KT; PerkinElmer). Finally, DAPI was applied for counterstaining and slides were mounted using glycerine.

Slides were scanned with the PerkinElmer Vectra (Vectra 3.0.5) and analyzed by the InForm Advanced Image Analysis software (inForm 2.3.0; PerkinElmer). The software was trained for specific algorithms (phenotyping, cell segmentation, tissue segmentation, etc.) before used for the test sample, and single stained slides were used to build a spectral library for unmixing. For surgical specimens more than 10 fields of view in 200× magnification of each tissue slide were selected to calculate the count, density, percentage of the positive cells. Percentage calculated by number of stained cells in the total number of cells was used for analyses. No data was obtained from slide 3 of puncture tumor tissue because of tissue falling during staining process and no other slide was available.

Targeted gene expression assay

Expression of immune-related genes were analyzed by the Oncomine^{TM} Immune Response Research Assay (Cat. No. A32881, ThermoFisher Scientific, USA), which quantitatively evaluate the expression of 400 genes involved in tumor-immune interactions. RNA from FFPE tissue sample was extracted, quantified, and reversed using the recommended kits. RNA libraries were prepared with a multiplex primer pool and were sequenced on the Ion PGMTM Systems following manufacture's protocols. Gene expression level was initially quantified as reads per million (RPM) by the Torrent Suite^{TM} immuneResponse RNA plugin. Then RPM of each gene was normalized as nRPM by an internal control sample, and log2 transformed nRPM was used for analyses

Plasma protein multiplex immunoassay

Peripheral blood samples were collected from the patient prior to each cycle of immunotherapy and the radical surgery, and four weeks after surgery as shown in figure 1C. 59 proteins in plasma were simultaneous detected by two ProcartaPlex panels with sandwich ELISA based multiplex immunoassays, including Cytokine/Chemokine/Growth Factor 45-Plex Human ProcartaPlex™ Panel 1 and Immuno-Oncology Checkpoint 14-Plex Human ProcartaPlex™ Panel 2, as per the manufacturer's instructions (ThermoFisher, USA).

Figure S1

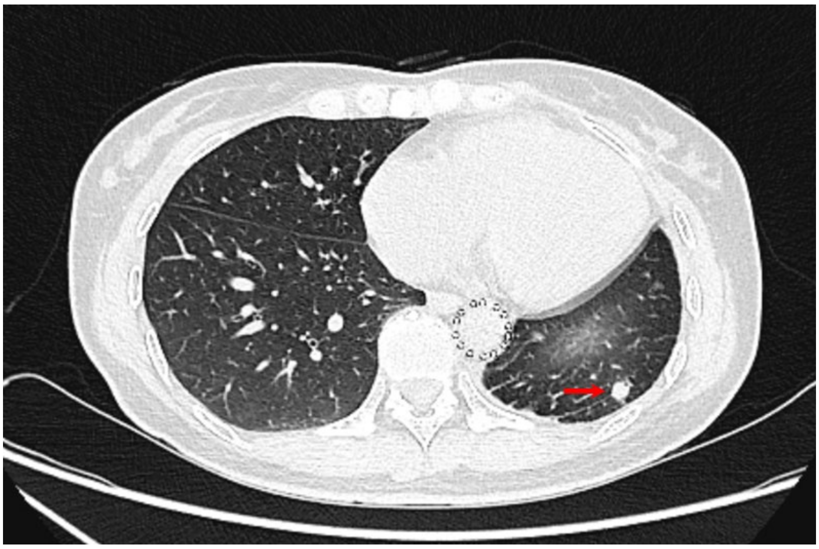

CT image showed the lung metastasis 8 months after surgery. The red arrow indicates the metastatic tumor.

Figure S2

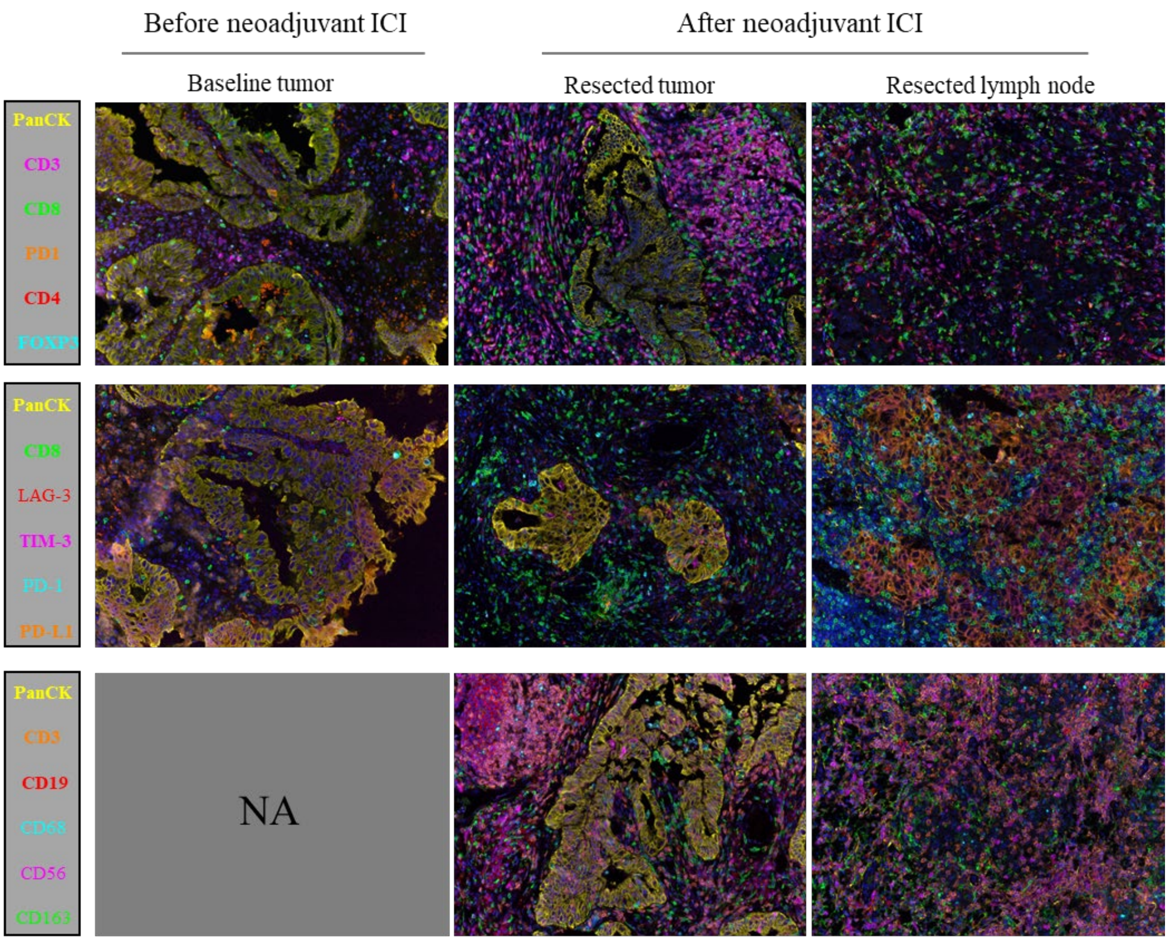

NA, not available. ICI, immune checkpoint inhibition. Original magnification, 200×

Figure S3

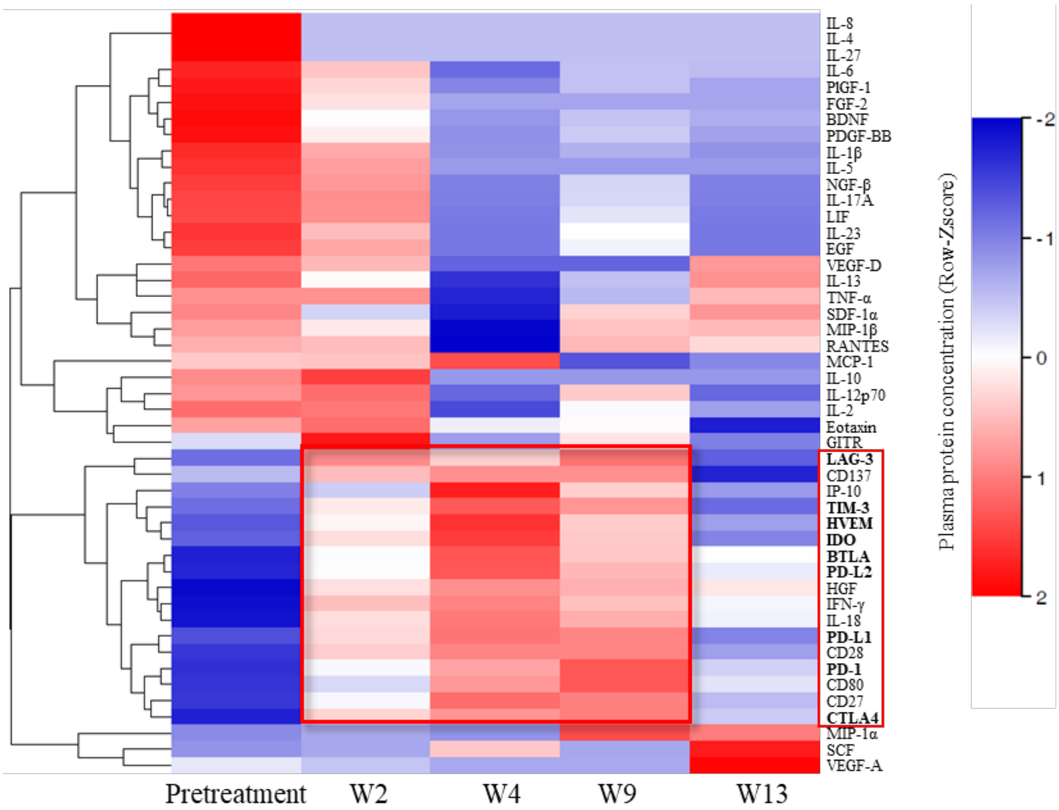

The red box shows that most of the costimulatory and checkpoint proteins are increased during neoadjuvant immunotherapy. Inhibitory checkpoint proteins are highlighted in bold. W2, W4, prior to each cycle of immunotherapy; W9, prior to the radical surgery; W13, four weeks after surgery.

Table S1

	CD3	CD4	CD8	CD19	CD68	CD56	FOXP3	PD-L1	PD-1	TIM-3	LAG-3	CD163
**Mean percentage (%)**												
**Baseline tumor**	3.1	0.1	4.2	-	-	-	5.5	3.4	5.2	0.0	0.3	-
**Resected tumor**	42.9	35.9	18.1	22.4	5.0	2.6	12.2	7.6	7.2	5.2	1.9	3.9
**Resected LN**	14.7	33.8	17.7	7.5	0.1	4.1	4.9	22.1	10.0	1.2	1.2	28.1
**Mean cell density (cell/mm2)**												
**Baseline tumor**	218.5	8.7	269.5	-	-	-	387.4	171.9	339.4	0.0	14.6	-
**Resected tumor**	3704.2	3161.5	1476.9	1905.4	411.1	217.6	1076.0	591.4	588.6	397.9	150.6	319.6
**Resected LN**	1187.4	2327.3	1260.4	624.6	8.1	337.9	344.8	1632.4	762.2	84.5	88.2	2047.8
**Mean cell number**												
**Baseline tumor**	75	3	93	-	-	-	133	59	117	0	5	-
**Resected tumor**	1219	1085	509	593	124	63	369	203	202	137	52	97
**Resected LN**	393	799	433	200	3	111	118	560	262	29	30	678

The data is the mean values of positive cells per field of view. The resected tumor and lymph node had more than 10 fields of view, while the baseline tumor had no more than two because of the small size. LN, lymph node.

Table S2

Primary antibody	Provider	Catalogue number	Clone	Host species	Concentration	Primary antibody incubation	TSA fluorophore (nm)
Panel 1							
FoxP3	Abcam	ab20034	236A/E7	Mouse	1:400	37°C 1 Hour	650
CD4	Zsbio	ZM0418	UMAB64	Mouse	1:100	37°C 1 Hour	620
CD8	Zsbio	ZA0508	SP16	Rabbit	1:100	4°C Overnight	520
PD1	Zsbio	ZM0381	UMAB199	Mouse	1:100	37°C 1 Hour	570
CD3	Zsbio	ZM-0417	UMAB54	Mouse	1:50	37°C 1 Hour	690
Panck	Zsbio	ZM0069	AE1/AE3	Mouse	1:100	37°C 1 Hour	540
Panel 2							
CD8	Zsbio	ZA0508	SP16	Rabbit	1:100	4°C Overnight	520
TIM3	CST	CST45208S	D5D5R	Rabbit	1:100	37°C 1 Hour	690
PD1	Zsbio	ZM0318	UMAB199	Mouse	1:100	4°C Overnight	650
LAG3	Abcam	ab40466	17B4	Mouse	1:100	37°C 1 Hour	620
PDL1	CST	CST13684S	E1L3N	Rabbit	1:100	37°C 1 Hour	570
Panck	Zsbio	ZM0069	AE1/AE3	Mouse	1:100	37°C 1 Hour	540
Panel 3							
CD163	Zsbio	ZM0428	10D6	Mouse	1:200	37°C 1 Hour	520
CD68	Zsbio	ZM0060	KP1	Mouse	1:500	37°C 1 Hour	650
CD3	Zsbio	ZM-0417	UMAB54	Mouse	1:50	4°C Overnight	570
CD56	Zsbio	ZM0057	UMAB83	Mouse	1:50	37°C 1 Hour	690
CD19	Zsbio	ZM-0038	UMAB103	Mouse	1:100	37°C 1 Hour	620
Panck	Zsbio	ZM0069	AE1/AE3	Mouse	1:100	37°C 1 Hour	540

Abbreviations: TSA, tyramide signal amplification.
